# Proteinase 3-Antineutrophil Cytoplasmic Antibody (PR3-ANCA)-Associated Vasculitis: A Case Report

**DOI:** 10.7759/cureus.91582

**Published:** 2025-09-04

**Authors:** Angela Almeida, Patricia Brito, Pedro Miranda, Sandra Barbosa, Jorge Cotter

**Affiliations:** 1 Internal Medicine, Hospital Senhora da Oliveira, Guimarães, PRT

**Keywords:** anca-associated vasculitis, diffuse alveolar hemorrhage, pauci-immune glomerulonephritis, pr3-anca, rituximab

## Abstract

Antineutrophil cytoplasmic antibody (ANCA)-associated vasculitis (AAV) is a group of rare, heterogeneous, and potentially life-threatening diseases, typically characterized by their phenotype (microscopic polyangiitis, granulomatosis with polyangiitis, or eosinophilic granulomatosis with polyangiitis) but currently classified according to the type of antibody that is identified: proteinase 3 (PR3), myeloperoxidase (MPO), or negative.

A 55-year-old man presented with a two-week history of fever, myalgias, and odynophagia, and two days of pleuritic pain, cough, hemoptysis, and purpuric lesions on both inferior limbs. CT scan documented multiple lung nodules surrounded by ground-glass opacities, and laboratory tests showed increased erythrocyte sedimentation rate, acute kidney injury, microscopic hematuria, and non-nephrotic proteinuria. Additional tests revealed PR3-ANCA positivity. Diffuse alveolar hemorrhage was confirmed, and a kidney biopsy unveiled pauci-immune focal crescentic glomerulonephritis. After two weeks of glucocorticoids, he initiated rituximab, with a gradual, promising response.

Effective treatment is vital in chronic inflammatory diseases, such as AAV, with an impact on both organ damage and global prognosis, which is why this diagnosis should be considered in the presence of suspected clinical signs or symptoms, to achieve better therapeutic results.

## Introduction

Antineutrophil cytoplasmic antibody (ANCA)-associated vasculitis (AAV) is a group of rare, heterogeneous, and potentially organ and life-threatening multisystemic autoimmune small vessel vasculitides that include microscopic polyangiitis (MPA), granulomatosis with polyangiitis (GPA), and eosinophilic granulomatosis with polyangiitis (EGPA) [1-4]. This subgroup’s definition is based on clinical features, that may include constitutional symptoms and specific features of end-organ involvement [1,2], but genetic and other clinical findings suggest that these syndromes are better classified as proteinase 3 (PR3)-positive, myeloperoxidase (MPO)-positive, and, for EGPA, by the presence or absence of ANCA [4], with GPA being strongly associated with PR3-ANCA [1,4-7]. Virtually any tissue can be affected, and the presentation can range from a skin rash to fulminant multisystemic disease [8], but the upper and lower respiratory tract and kidneys are most usually and severely involved [2,4,6]. Kidney involvement, typically characterized by a pauci-immune necrotizing and crescentic glomerulonephritis [1,4,5,7,9,10], is particularly important because of its frequency (about 70%) and its impact on prognosis, with a very quick decline of renal function if untreated [9]. Generally, a two-phase treatment is recommended: induction of remission with a combination of glucocorticoids and another immunosuppressive agent, followed by maintenance therapy to maintain remission [2,4,5,10]. According to two randomized controlled trials [11,12], rituximab was as effective as conventional cytotoxic cyclophosphamide to induce remission in adults with severe GPA (combined with glucocorticoids) and possibly less toxic, becoming the drug of choice in specific cases. The optimal duration of glucocorticoid and maintenance treatment remains uncertain and should be tailored to each patient [10], but it is normally advised that maintenance therapy should continue for at least 24 months before being gradually withdrawn [2,8]. Longer duration of therapy should be considered in case of relapse or increased risk of relapse [2,4,5]. Since AAV is now considered to be a chronic disease, requiring long-term immunosuppressive therapy, attention must be given to the treatment-associated morbidity [12]. Additionally, even though the five-year survival is around 75% [4,5,8], the overall late mortality remains high due to infection, cardiovascular disease, or malignancy [4,8,13], thus all patients should be given prophylaxis against *Pneumocystis jirovecii* pneumonia and vaccine against pneumococcus and influenza virus, along with screening for cardiovascular and renal disease and osteoporosis prevention [4,8,9,13].

## Case presentation

**Figure 1:**
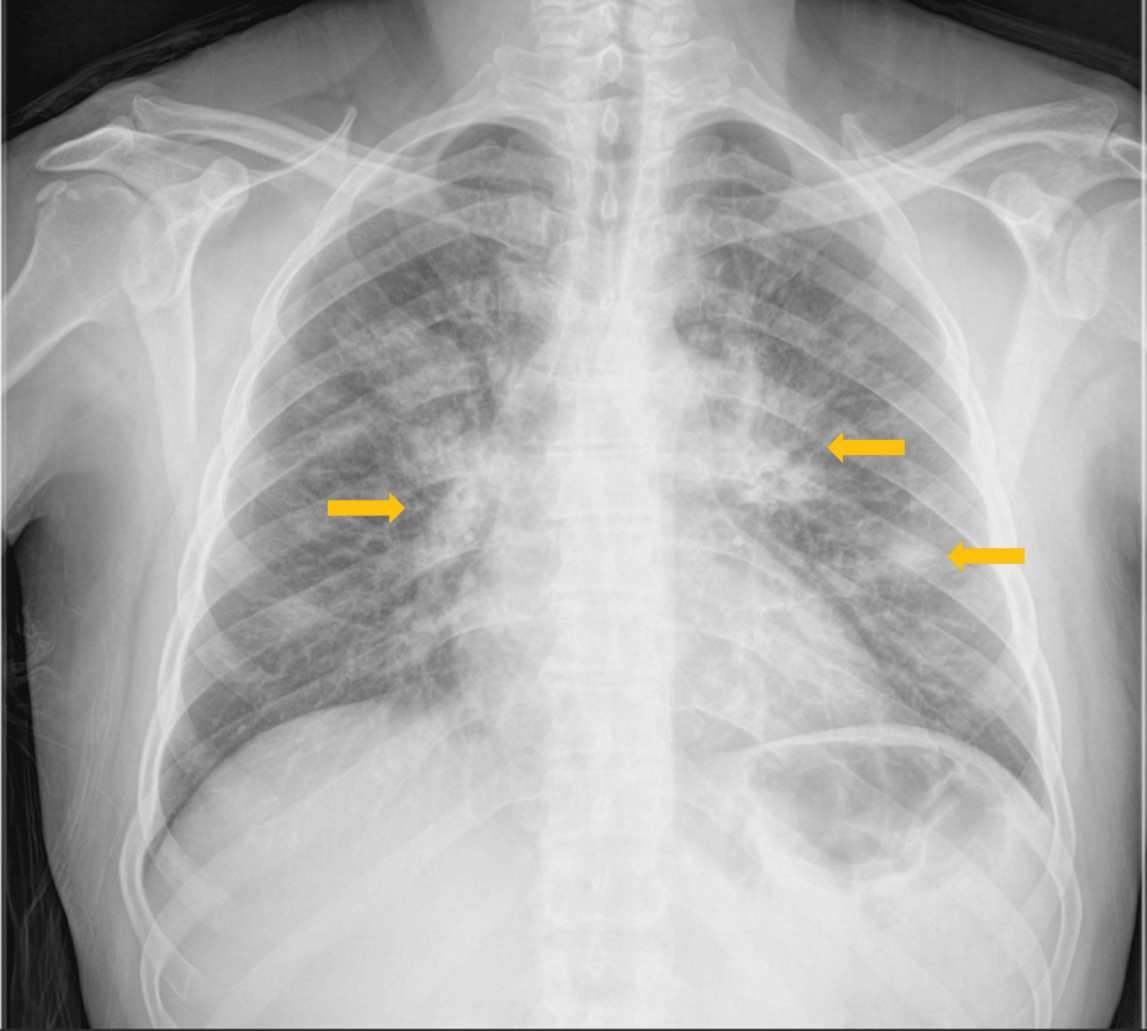
Initial chest X-ray that led to pneumonia diagnosis, showing bilateral diffuse opacities/infiltrates (yellow arrows).

A 55-year-old man with no occupational history or environmental exposures and a description of arterial hypertension and dyslipidemia, under no pharmacological therapy, presented to the emergency department (ED) with a two-week history of fever, myalgias, and odynophagia. Based on the laboratory data shown in Table 1 and an initial chest X-ray (Figure 1), he was diagnosed with pneumonia and discharged with a prescription of amoxicillin/clavulanate and azithromycin.

**Table 1:** Initial laboratory tests results that led to pneumonia diagnosis.

		Reference range
Hemoglobin (g/dL)	9.7	14.0 - 18.0
Leukocytes (cells/µL)	10700	4800 - 10800
Serum creatinine (mg/dL)	1.04	0.70 - 1.30
C-reactive protein (mg/L)	232.9	<3.0

**Figure 2:**
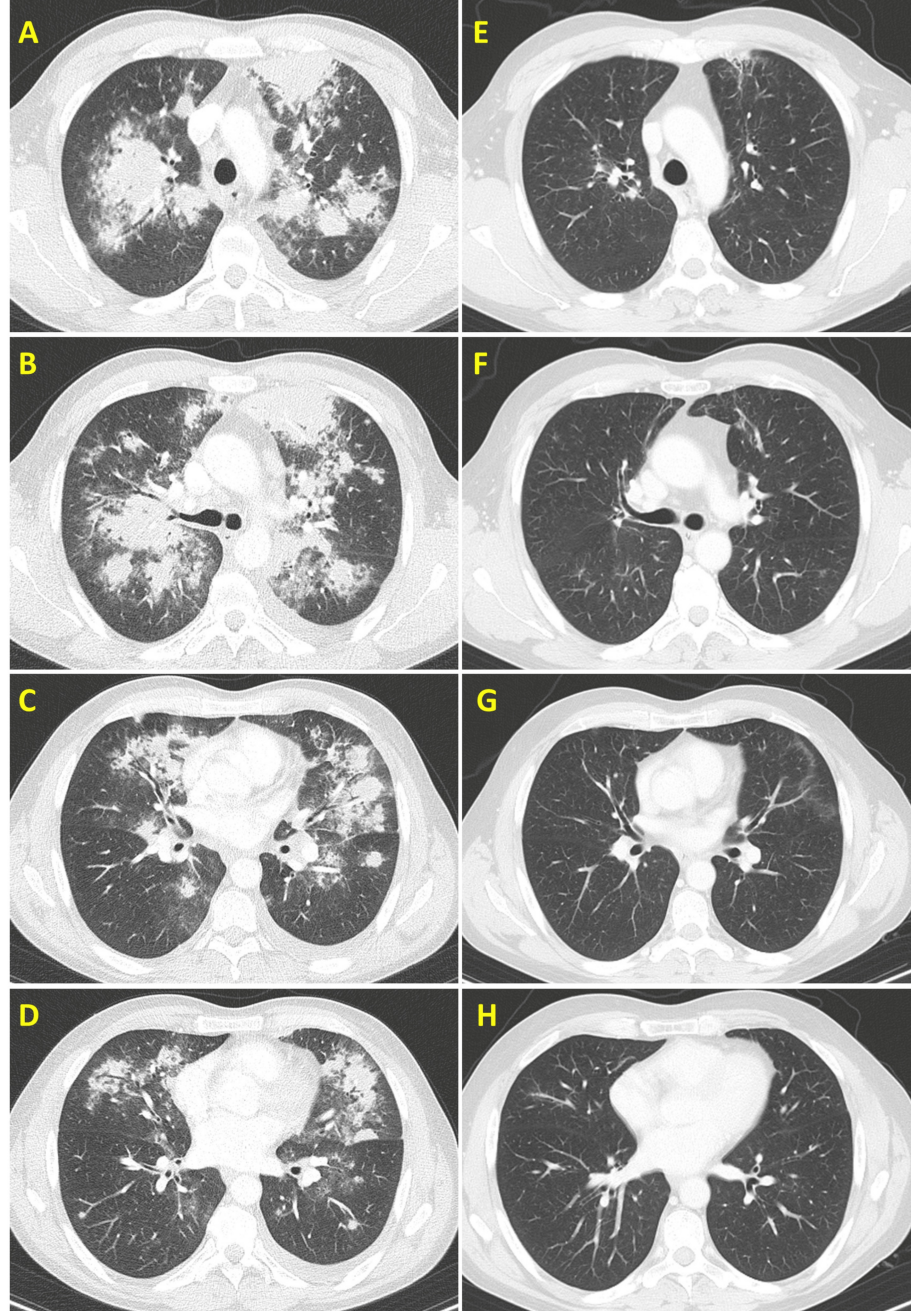
Lung involvement as shown in the emergency department CT scan (A-D) and after two years of treatment (E-H). Note the multiple lung nodules surrounded by ground-glass opacities, suggestive of alveolar hemorrhage. Comparative images show radiological improvement after induction of remission (A versus E, B versus F, C versus G, and D versus H).

After two days, he developed pleuritic pain, cough, hemoptysis, and purpuric lesions on both inferior limbs and returned to the ED. Initial laboratory tests showed mild anemia, leukocytosis, increased C-reactive protein and erythrocyte sedimentation rate, and microscopic hematuria (Table 2). Real-time PCR testing was negative for SARS-CoV-2, influenza A, influenza B, and respiratory syncytial virus. Chest computed tomography (CT) scan revealed multiple areas of nonspecific parenchymal lung opacities, pulmonary nodules, and ground-glass pattern (Figure 2), and he was admitted to the internal medicine department to undergo further investigation.

**Table 2:** Laboratory tests results at presentation versus after induction of remission.

Test	Admission	After induction of remission	Reference range
Hemoglobin (g/dL)	8.7	14.1	14.0 - 18.0
Leukocytes (cells/µL)	14500	8800	4800 - 10800
Platelets (cells/µL)	391000	194000	150000 - 250000
Serum creatinine (mg/dL)	2.6	1.5	0.70 - 1.30
C-reactive protein (mg/L)	347.7	1.0	<3.0
Erythrocyte sedimentation rate (mm)	102	21	0 - 12
Ferritin (µg/mL)	4658.1	403	22 - 322
Urine red blood cells (cells/high-power field)	74	1	<5
24-hour urine protein (g/24 h)	1.5	0.187	<0.149

Despite being under broad-spectrum antibiotics, his clinical condition worsened within the first days, with type 1 respiratory failure and acute kidney injury with non-nephrotic proteinuria (Table 2). Specific tests detected PR3-ANCA, and it was decided to introduce high-dose glucocorticoid, including five days of methylprednisolone 1 g/day, followed by prednisolone 1 mg/kg/day, while waiting for bronchoscopy and kidney biopsy. Bronchoscopy revealed multiple endobronchial nodules, whose biopsy documented areas of fibrin with extravasation of erythrocytes, and bronchoalveolar lavage fluid exposed progressively hemorrhagic return, confirming diffuse alveolar hemorrhage. Kidney biopsy unveiled pauci-immune focal necrotizing and crescentic glomerulonephritis. The patient was also observed by an otorhinolaryngologist, who documented paranasal sinusitis, nasal crusting, and nasal septum deviation.

After two weeks of high-dose glucocorticoid, he initiated induction of remission with rituximab as well as cotrimoxazole as a prophylactic agent.

Currently, glucocorticoid tapering is proceeding according to recommendations [2], along with the rituximab protocol [2], without major complications to be described. The patient is now asymptomatic and presents laboratory and radiological improvements (Table 2 and Figure 2).

## Discussion

AAV is relatively rare, and some clinical findings are nonspecific, leading to an estimated diagnostic delay greater than six months in about one-third of patients [4,8,13], which can impact patient outcome. However, if promptly recognized, AAV can respond well to immunosuppressive drugs, with a similar prognosis to other chronic inflammatory diseases, according to the degree of established organ damage prior to diagnosis [3]. In this case, diagnosis was established within three weeks due to the concurrent presentation of microscopic hematuria, non-nephrotic proteinuria, and multiple lung nodules surrounded by ground-glass opacities.

Some studies suggest that PR3-ANCA+ patients may respond better to rituximab (versus cyclophosphamide) [4,5,8], which is why it was the chosen immunosuppressive agent in this case. PR3-ANCA+ patients are known to have a higher risk of relapse than MPO-ANCA+ patients, especially if there is upper or lower respiratory involvement [4,5,8], but several other factors alter prognosis such as older age, delayed diagnosis, severity of renal dysfunction and histological pattern of kidney involvement [5,10] and presence of alveolar hemorrhage [5] or ENT disease [5]. Therefore, we recognize that this patient has an increased risk of relapse because of his disease phenotype and presentation, despite his great response to induction therapy, as shown by laboratory test results and CT-scan images (Table 1 and Figure 1). On this note, maintenance therapy aims to prevent relapses, minimize the risk of comorbidities and drug toxicity, and manage organ damage, and many patients need extended low-dose glucocorticoids even if treated with rituximab [5,10]. For that reason, the optimal duration of treatment with these agents remains uncertain, with some studies suggesting 24-48 months of therapy regardless of disease phenotype [5,10].

## Conclusions

AAV is a rare multisystemic autoimmune disease with a broad clinical spectrum, ranging from non-specific clinical findings to life-threatening situations. As a result, AAV can be a diagnostic challenge demanding a very careful and systematic approach to establish a correct and sharp diagnosis. In this case, we could properly diagnose PR3-ANCA vasculitis and start suitable therapy to improve the patient’s condition and prevent future complications. Considering that most patients need long-term immunosuppressive therapy, the medical team should be aware of possible treatment-associated adverse events, since they are an important cause of morbidity and mortality. To this point, this patient had a promising complication-free response to rituximab in combination with glucocorticoid, but appropriate medical monitoring is required to achieve a favorable outcome.
